# A risk prediction model for medical conflict in emergency departments

**DOI:** 10.3389/fpubh.2026.1734894

**Published:** 2026-01-29

**Authors:** Fengjiao Gu, Junlin Huang, Yingqian Zhang, Jun Zeng, Hua Jiang, Michael Maes, Li Gou

**Affiliations:** 1 Sichuan Provincial Center for Mental Health, Sichuan Provincial People’s Hospital, School of Medicine, University of Electronic Science and Technology of China, Chengdu, China; 2 Department of Information Center, Sichuan Provincial People’s Hospital, School of Medicine, University of Electronic Science and Technology of China, Chengdu, China; 3 Key Laboratory of Psychosomatic Medicine, Chinese Academy of Medical Sciences, Chengdu, China; 4 Department of Institute for Emergency and Disaster Medicine, Sichuan Provincial People’s Hospital, University of Electronic Science and Technology of China, Chengdu, China; 5 Department of Psychiatry, Medical University of Plovdiv, Plovdiv, Bulgaria; 6 Research Institute, Medical University of Plovdiv, Plovdiv, Bulgaria; 7 Research and Innovation Program for the Development of MU–PLOVDIV (SRIPD-MUP), Creation of a Network of Research Higher Schools, National Plan for Recovery and Sustainability, European Union–NextGenerationEU, Medical University of Plovdiv, Plovdiv, Bulgaria; 8 Department of Psychiatry, Faculty of Medicine, Chulalongkorn University, Bangkok, Thailand; 9 Kyung Hee University, Seoul, Republic of Korea; 10 Department of Nursing ResearchSu Center, Sichuan Provincial People’s Hospital, University of Electronic Science and Technology of China, Chengdu, China

**Keywords:** doctor-patient relationship, emergency departement, medical conflict, prediction model, risk assesssment

## Abstract

**Objective:**

This study aims to identify and analyze patient-related risk factors that lead to medical conflict in emergency departments and to build a patient-related risk prediction model, which can serve as a tool for medical professionals to prevent medical conflict in emergency departments.

**Background:**

At present, the research on the countermeasures of medical conflict in emergency departments mainly focuses on post-event coping strategies and rarely addresses the early warning of conflict occurrence.

**Methods:**

Through a retrospective analysis of medical conflict events in the emergency pre-examination and rescue areas, 105 conflict cases and 525 non-conflict cases were collected. Univariate analysis, neural network analysis and support vector machine (SVM) were performed to analyze patient-related risk factors in medical conflict, thereby constructing a prediction model.

**Results:**

Neural network analysis yielded an accuracy of 96.4% (sensitivity: 91.2%, specificity: 97.7%) and an area under the ROC curve of 0.966. The order of importance of risk factors included in the prediction model is in descending order of importance: waiting time; patient non-compliance to the treatment process; patient’s skepticism toward care; number of caregivers; medical professionals’ failure to respond to patients’ needs; and history of psychosomatic diseases. The SVM utilizing 10-fold cross-validation demonstrated an accuracy of 96.4% in independent testing samples, with a sensitivity of 91.2% and a specificity of 97.2%.

**Conclusion:**

This model may accurately predict the occurrence of medical conflicts in emergency departments with high accuracy, providing a theoretical basis for preventing medical conflicts.

**Implications for management:**

We have developed a risk prediction model to help emergency medical professionals identify factors that may contribute to preventing medical conflict, reducing conflict events, and enhancing the doctor-patient relationship.

## 1 Background

Doctor-patient conflict refers to the tension or dispute between doctors and patients during diagnosis, treatment, or care, resulting from differing perceptions about the process, attitude, or outcome (1). In recent years, amid intensifying reforms in the medical and healthcare systems, patients’ increasing demand for services and heightened awareness of their rights have led to a rise in doctor-patient disputes. The Chinese Hospital Association reports an average of 27 violent incidents involving doctors per hospital annually in China (2). A 2019 survey indicated that 33.48% of doctors experienced disputes, 20.86% disturbances, 48.52% verbal violence, and 5.84% physical violence (3). In cases of hospital violence, the most common perpetrators are patients’ relatives (58.2%), followed by the patients themselves (38.2%).

The emergency department, which often deals with a high volume of critical cases with complex conditions, is particularly susceptible to medical conflict. According to Alpha Case Library, in 2021, there were 10,746 medical liability disputes, with outpatient and emergency departments recording the highest numbers. Globally, around 50% of emergency nurses face physical violence, and 80% encounter psychological violence (4). A survey from 2013 to 2021 on workplace violence against Chinese health professionals revealed that the emergency department had the highest incidence of violence in healthcare (18.9%) (5). 80.7% of emergency medical professionals in China experienced workplace violence. Workplace violence against medical staff in China is a widespread problem that has negative impacts on medical service delivery (6), while Vezyridis et al. (7) noted that 76.2% of emergency medical professionals faced abuse from patients within a year. Existing studies have identified that workplace violence can significantly lead to depression. Medical conflict not only exhausts patients and causes them mental distress (1) but also increases psychological stress and job burnout among medical professionals, severely damaging the healthcare profession’s reputation and social status (8, 9).

Research indicates that proactive analysis of risk factors impacting medical quality and patient safety in diagnosis and treatment allows for early intervention, effectively reducing medical disputes and fostering an excellent clinical environment (9). Thus, early warning of medical conflict is vital. Previous studies have identified risk factors for medical conflict, including inadequate patient-doctor communication (10, 11), misunderstandings regarding medical procedures (10), and a lack of empathy. However, comprehensive analyses of these factors are still lacking.

A prediction model, which assesses multiple core factors to anticipate specific outcomes, can be instrumental in identifying and mitigating risks (12). Scholars have recently developed risk prediction models for acute medical events like heart failure, cardiovascular disease, and cancer (13–15). Building on this foundation, our study analyzes patient-related risk factors in emergency department medical conflicts. By constructing and validating a predictive model, we aim to provide a quantitative tool for assessing the likelihood of medical conflicts in emergency settings.

## 2 Methods

### 2.1 Setting

The research object is emergency patients in a large-scale tertiary hospital, which has 4,300 beds and receives approximately 240,000 emergency patients annually.

### 2.2 Design

#### 2.2.1 Sample size

This study is a retrospective design, and the sampling method employed was time-matched random sampling. Conflict cases in the emergency department from June 2024 to January 2025 were collected. The number of predictors in the prediction model established in this study was expected to be 10 or fewer. According to the rule that the number of events is 10 times the number of predictors, at least 100 events (conflict cases in this study) would be required. Given that the conflict is a rare event, considering efficiency and cost, we excluded all non-conflict patients during the target period. This study used the case–control study method for sample size selection. We identified the observed medical conflict events as the case group and selected patients without conflict in the same period as the control group, based on a ratio of 1:5. This resulted in a minimum sample size of 100 conflict cases and 500 non-conflict cases. The sample collected comprised 105 conflict cases and 525 control cases, totaling 630 cases, which met the sample size requirements. Inclusion criteria: (a) The patient is ≥ 18 years old; (b) The whole treatment was completed in the emergency department. Exclusion criteria: incomplete clinical data. The retrospective study was approved by the Ethics Committee of Sichuan Provincial People’s Hospital [Ethics (Research) 2025–734].

#### 2.2.2 Data collection

Based on literature research and expert consultation, we created the “record of conflict risk factors” and the “record of conflict events.” The “record of conflict risk factors” includes risk factors related to patients’ general information, health conditions, and doctor-patient interactions. The “record of conflict events” records the time, place, type, and handling of the conflict.

In this study, a patient-doctor conflict was identified if any of the following occurred between the healthcare provider and the patient: verbal conflict (e.g., quarreling, verbal abuse, arguments, complaints), physical conflict (e.g., hitting, pushing, beating), or other forms of conflict (such as vandalism). The assessors were other medical staff working in the emergency department at the time the conflict occurred.

Subjective measures such as patient distrust, perceived delays in medical care, and non-compliance were extracted from the patient satisfaction assessment forms completed during the visit. Other quantitative data were retrieved by our researchers, who were blinded to the study outcomes, from medical records, emergency incident reports, and related documentation.

#### 2.2.3 Statistical methods

This study used SPSS 26.0 for data analysis. Frequency analysis was performed on the basic information, and the independent sample chi-square test was used for univariate analysis. Neural network analysis was performed for constructing a risk prediction model. We employed a multilayer perceptron neural network model utilizing several risk factors as input variables, and categorizing the output as either “risk” or “no risk” for conflict. An automatic feedforward network model utilized up to eight nodes, incorporating one or two hidden layers. The termination criterion was defined by a consecutive step that failed to further decrease the error term. The neural network model underwent training for a maximum of 250 epochs in a batch training session. The error and misclassification rate as well as the area under the ROC curve, were calculated, and the models’ predictive validity was assessed using accuracy, with sensitivity and specificity calculated in an independent holdout sample. The relevance and relative prominence of the input variables were assessed and depicted in an importance chart.

We additionally employed the support vector machine (SVM) due to its margin-based, regularized classification capabilities, hence complementing neural networks. Convergent performance across distinct algorithms (SVM versus NN) enhances the robustness and reproducibility of the classification outcomes. SVM was performed using STATISTICA 14.0, employing a Type 1 classification SVM with a radial basis function (RBF) kernel. Models were trained on 68% of the dataset and assessed on an independent 32% test set, utilizing 10-fold cross-validation within the training set for hyperparameter tuning. All variables were scaled prior to modeling.

## 3 Results

### 3.1 Description of medical conflict events

A total of 105 medical conflict cases were collected, of which 78 occurred in the daytime, and 27 occurred at night. Twenty-one conflict cases occurred on weekends, and 84 on weekdays. There were 56 conflict cases in the pre-examination and triage areas, 41 in the resuscitation rooms, and eight in the consulting rooms. Regarding the medical professionals, 80 conflict cases involved nurses and 16 involved doctors. The attackers were caregivers in 67 cases, patients in 31 cases, and both caregivers and patients in 7 cases. There were 49 cases with female attackers, 53 with male attackers, and 3 with both male and female attackers. See Table 1 for a summary of the conflict events.

**Table 1:** Characteristics of medical conflict events (*n* = 105).

Variable	*n*	Percentage
Verbal violence		
Quarrel	41	34.45%
Verbal abuse	39	32.77%
Disputation	9	7.56%
Complain	30	25.21%
Physical violence		
Hitting	7	36.84%
Pushing	9	47.37%
Beating	3	15.79%
Other violence		
Damage to public property	1	12.50%
Filming without permission	6	75%
Spitting	1	12.50%
Consequences of conflict		
Minor abrasions of the medical professionals	5	8.62%
Soft tissue injury of medical professionals	2	3.45%
Fracture or organ injury of medical professionals	1	1.72%
Disturbance to other patients’ diagnosis and treatment	5	8.62%
Disturbance to regular services	45	77.59%
Conflict handling		
Private settlement	57	54.29%
Involvement of the department’s directors	26	24.76%
Involvement of the supervising organization	1	0.95%
Involvement of the hospital’s dispute resolution office	2	1.90%
Involvement of the police	5	4.76%
Involvement of the security	14	13.33%

### 3.2 Description of patients with conflict events

This study included 105 patients with conflict events, including 58 males and 47 females. See Table 2 for a summary of the patients’ information.

**Table 2:** Characteristics of patients with conflict (*n* = 105)

**Variable**	**Category (assigned value)**	***n***	**Percentage**
**Gender**	Male (0)	58	55.24%
	Female (1)	47	44.76%
**Age**	< 35 (1)	6	5.71%
	35-59 (2)	16	15.24%
	> 60 (3)	83	79.05%
**Waiting time**	< 10 minutes (1)	72	68.57%
	10-30 minutes (2)	20	19.05%
	> 30 minutes (3)	13	12.38%
**Stage of the medical process**	Initial diagnosis (0)	21	20%
	Follow-up (1)	84	80%
**Way of admission**	Self-admission (0)	75	71.43%
	Ambulance (1)	30	28.57%
**Symptoms that may lead to harming or misunderstanding others**	Delirium (1)	11	10.48%
	Delusion (1)	2	1.90%
	Confusion (1)	2	1.90%
	Disorientation (1)	1	0.95%
**Triage level**	Level 1 (1)	51	48.57%
	Level 2 (2)	13	12.38%
	Level 3 (3)	39	37.14%
	Level 4 (4)	2	1.90%
**History of psychosomatic diseases**	Yes (1)	7	6.67%
**Caregivers**			
**With Caregivers**	Yes (1)	96	91.43%
**Drinkers**	Yes (1)	1	0.95%
**Gender of caregivers**			
	Only males (0)	43	40.95%
	Only females (1)	43	40.95%
	Males and females (2)	19	18.10%
**Number of caregivers**			
	≥ 3	46	43.81%
	2	43	40.95%
	≤ 1	16	15.24%

Note: The “triage level” is based on the emergency department triage.

### 3.3 Univariate analysis of risk factors of medical conflict

A total of 105 conflict cases and 525 non-conflict cases were included in this study. Univariate analysis showed that there was no significant between-group difference in the gender of patients, way of payment, type of conditions, whether there were symptoms that might lead to harming or misunderstanding others, gender of caregivers, and whether there were precipitants (*p* > 0.05). However, statistically significant differences were found in waiting time, age, stage of the medical process, way of admission, triage level, history of psychosomatic diseases, caregivers (number of caregivers), and doctor-patient interaction (medical professionals’ failure to respond to patients’ needs timely due to heavy workload; patient skepticism toward treatment and care; patient non-compliance to diagnosis and treatment) (*p* < 0.05). See Table 3 for a summary of the results.

**Table 3:** Logistic regression analysis of risk factors of medical conflict

**Risk factor**	**β**	**Standard error**	**Wald**	***df***	***p***	**OR**	**95% confidence interval**	
							**lower limit**	**upper limit**
Waiting time	–5.107	0.983	27.021	1	<0.001	0.006	0.001	0.042
Stage of the medical process	–0.126	0.705	0.032	1	0.86	0.881	0.221	3.509
Way of admission	–0.421	0.736	0.328	1	0.57	0.656	0.155	2.777
Triage level History of psychosomatic diseases	–0.607 –5.834	0.303 1.316	4.006 19.645	1 1	0.045 <0.001	0.545 0.003	0.301 0	0.987 0.039
Number of caregivers	1.653	0.407	16.522	1	<0.001	5.221	2.353	11.585
Medical professionals’ failure to respond to patients’ needs timely due to heavy workload	–5.483	0.793	47.816	1	<0.001	0.004	0.001	0.02
Patient skepticism toward treatment and care	–5.936	0.745	63.412	1	<0.001	0.003	0.001	0.011
Patient non-compliance with the diagnosis and treatment process	–6.986	0.918	57.874	1	<0.001	0.001	0	0.006

### 3.4 Neural network analysis on risk factors of medical conflict

**Figure 1:**
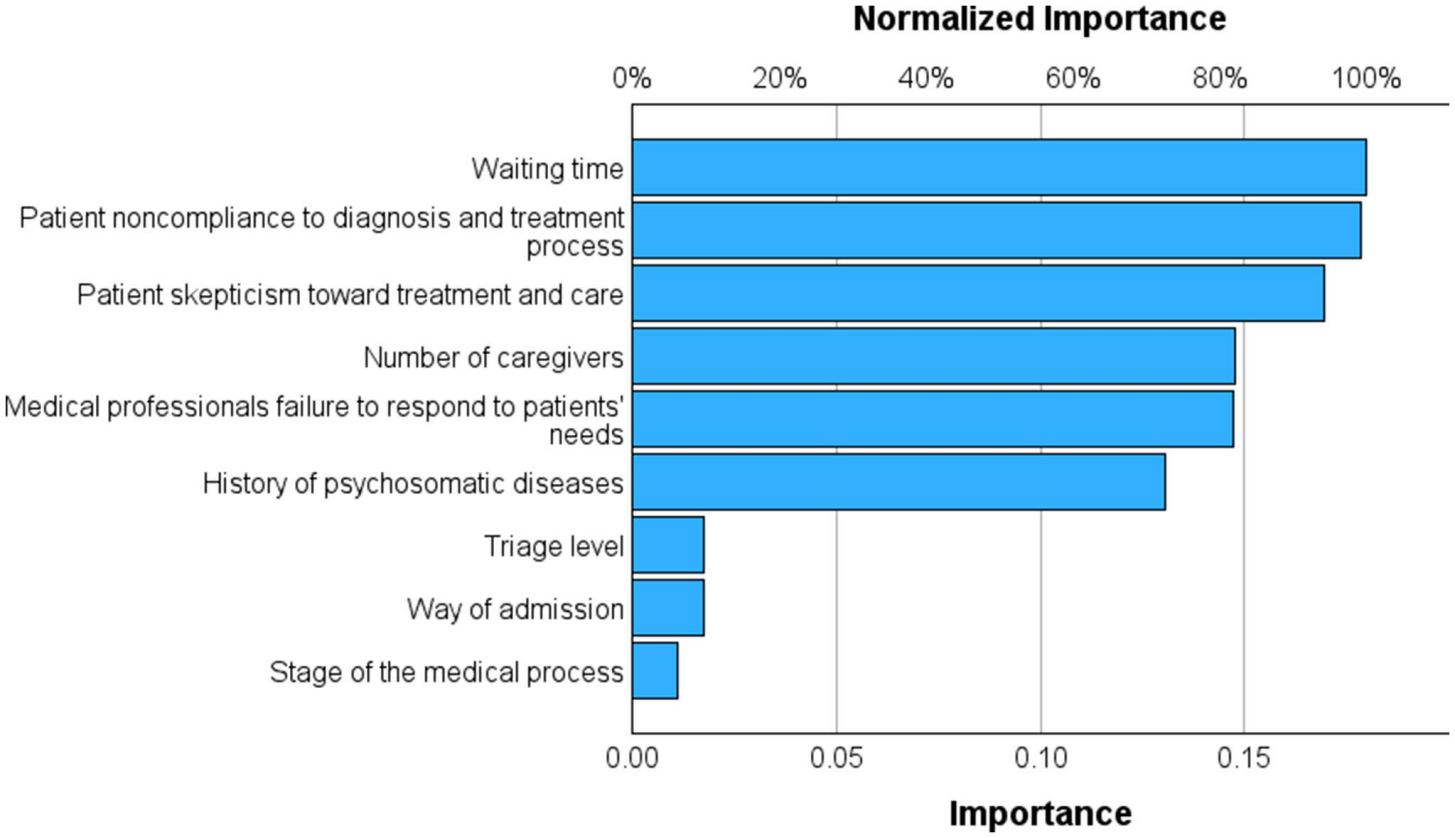
The risk factors of medical conflict analyzed by a neural network.

**Figure 2:**
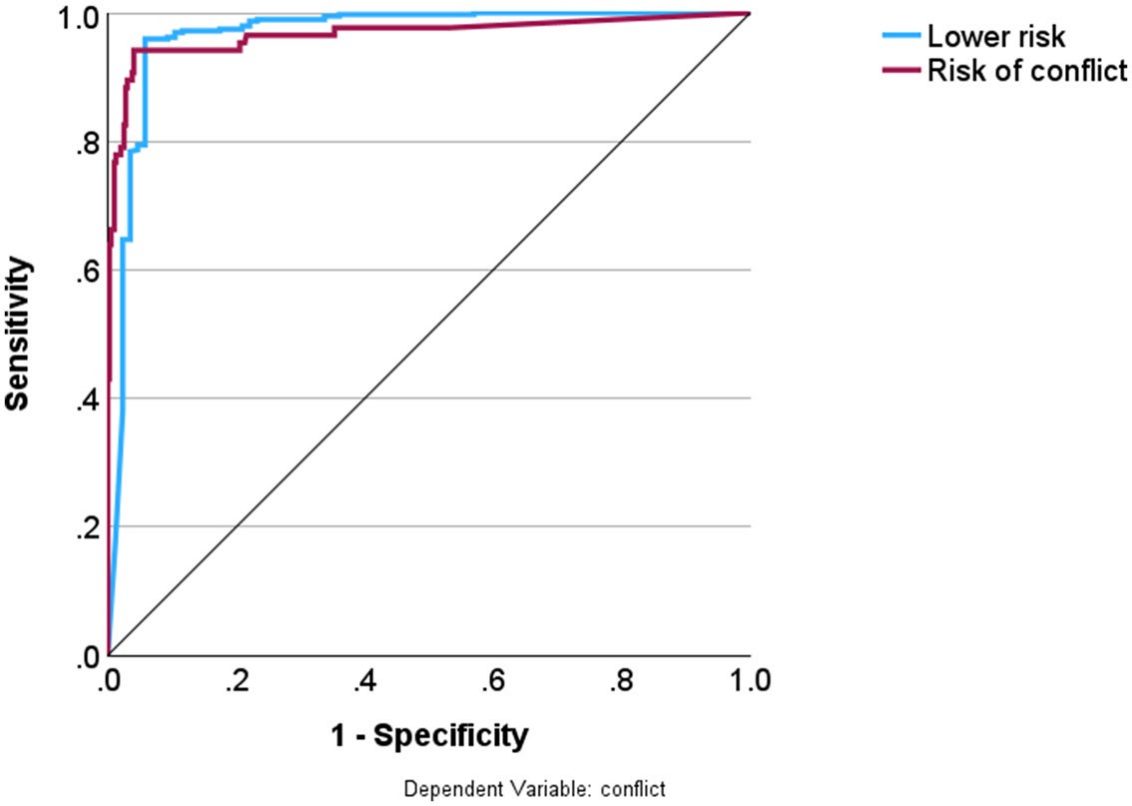
Predictive performance of the validity of the prediction model.

The final neural network model utilized the hyperbolic tangent as the activation function for the hidden output layers and the identity function for the output layer. Training was performed using two hidden layers, the first comprising five units and the second four units. The characteristics of the NN model are shown in ESF, including parameter estimates (Supplementary Table 1), neural network diagram (Supplementary Figure 1), model summary (Supplementary Table 2), and predicted versus observed chart (Supplementary Figure 2). During the training phase, the sum of squares error term was reduced from 14.425 to 4.626, indicating improved trend generalization by the model. Moreover, the model seems to have effectively minimized overfitting, as evidenced by the consistent percentage of incorrect predictions throughout training (5.2%), testing (3.2%), and holdout (2.7%) samples. These results indicate consistent, but not perfect, discrimination between the two groups. Figure 1 illustrates the importance chart, highlighting the significance of the key input variables. The model primarily recognized waiting time; patient non-compliance to the treatment process; patients’ skepticism, number of caregivers, medical professionals’ failure to respond to patients’ needs, and history of psychosomatic diseases as the most important predictors, followed at a distance by patient skepticism toward the cost; history of psychosomatic diseases; and number of caregivers. The cross-validated precision of the model (using a holdout sample) showed a predictive accuracy of 97.3%, with a sensitivity of 94.7% and a specificity of 97.7%. Figure 2 shows that the AUC of the ROC curve was 0.966.

The SVM model detected 76 support vectors, comprising 36 from the no-risk category and 40 from the risk category. The optimized model parameters were C = 10.0 and *γ* = 0.11111, employing an RBF kernel. The resulting decision function constant was 3.126763, signifying stable separation between classes. Model training attained an aggregate accuracy of 94.9%. In the independent test set, the model achieved an accuracy of 96.4%. The classification performance on the test set demonstrated a sensitivity of 91.2% and a specificity of 97.7%.

## 4 Discussion

### 4.1 Risk factor analysis of the prediction model

The results of the neural network analysis showed the importance of risk factors in predicting possible conflict: waiting time; patient non-compliance to the treatment process; patients’ skepticism, number of caregivers, medical professionals’ failure to respond to patients’ needs, and history of psychosomatic diseases.

Waiting time refers to the period a patient waits before their consultation. Waiting time was identified as the secondary risk factor. Prolonged waiting in emergency departments frequently triggers global dissatisfaction and conflicts among patients and their families. Our research results once again confirm this. Our results are similar to those reported by Vezyridis (16), who noted that patients in the emergency department considered their conditions urgent and believed they should receive comprehensive emergency treatment immediately. A longer waiting time, more severe conditions, and higher patient urgency for medical treatment increase the likelihood that the patient will be dissatisfied with the diagnosis and treatment process, leading to conflicts with medical professionals.

The second most important risk factor is “patient non-compliance to the diagnosis and treatment process”. It is related to two facts. First, in some hospitals, payment procedures, tests and examinations, result communication, and follow-ups are cumbersome, leading to patient drop-out and even conflict in emergent situations. Second, patients in the emergency department have severe conditions, and the pain makes them hope to get immediate treatment from medical professionals; when registration, triage, payment, or any other process does not go smoothly, they consider the medical process complicated and thus show poor compliance toward the diagnosis and treatment process, resulting in conflict, patients have high expectations of medical technology, while effective communication between doctors and patients is lacking (2). The verbal and nonverbal actions embodied in the doctor-patient interaction form the basis for establishing a harmonious relationship between the doctor and patient.

This study identified “patients’ noncompliance toward diagnosis, treatment, and care as the significant risk factor. This finding suggests that patients’ distrust and negative perceptions constitute the primary catalyst for conflicts. In the high-pressure setting of the emergency department, inadequate communication or a failure to meet patient expectations can readily trigger an eruption of such distrust. The reason may be that patients lack sufficient knowledge of emergency care, assuming they should receive immediate care and treatment in the emergency department. In fact, about 50% of the patients in the emergency department of a general hospital are non-emergency patients. Additionally, some patients or caregivers may not fully understand emergency treatment and hold overly high expectations for its effects. Consequently, patients may question the diagnosis, treatment, or care measures, which can potentially result in conflict.

Our study also indicated that a higher number of caregivers is related to a higher probability of conflict. It is due to caregivers’ concern about the patient’s condition. However, it could also result in an increased number of disputes and collective emotional outbursts, thereby elevating management challenges and the risk of conflict. A Chinese proverb states, “There is safety in numbers.” The caregivers will feel “impeccable” if there are many of them, and even slight dissatisfaction may make them prone to violent behaviors.

Our study also identified “medical professionals failure to respond to patients’ needs” as another significant contributor to conflicts. The emergency department is always busy with a heavy workload and high pressure. In a state of long-term mental tension, medical professionals may be irritable and impatient during work. They may sometimes fail to meet patients’ needs promptly, resulting in conflict.

In addition, patients with a history of psychosomatic diseases (of any level) have a potential risk of conflict with others and are prone to anxiety and irritability. Therefore, the history of psychosomatic diseases is one of the critical factors leading to medical conflict. Patients with this medical history may exhibit heightened anxiety and sensitivity to physical symptoms or pre-existing difficulties with emotional regulation, thereby increasing their susceptibility to conflicts.

### 4.2 Prediction effect of the model

Convergent performance of both neural network and SVM in independent holdout and testing samples (accuracy around 96–97%) suggests that our classification results may be reproducible. The prediction model can play a crucial role in the early detection of medical conflicts in emergency departments, enabling medical professionals to identify patients at risk of conflict and implement preventive measures and interventions in advance, thereby reducing the likelihood of medical conflicts. It has significant value for clinical practice.

### 4.3 The prediction model’s significance for clinical practice

In the emergency department, patients’ conditions are urgent, complex, and diverse, and patients and their families have an urgent need for medical treatment, making it prone to medical conflict. The study by Yang reported that the incidence of conflict in surgical emergencies was as high as 19%, and emergency services workers run the greatest risk of violence (17). The medical conflict significantly negatively impacts patients, family members, medical professionals, and hospitals. It disturbs hospitals’ regular medical services, damages medical professionals’ social reputation and image, causes significant psychological pressure to the professionals, and may even lead to violence. It harms the healthy development of the medical and healthcare industry and is not conducive to a harmonious society. Research has shown that acquiring relevant knowledge can prevent medical conflict (18). According to previous studies, the risk factors affecting the doctor-patient relationship primarily include a misunderstanding of medical behavior, overly high expectations for treatment, and inadequate or insufficient medical knowledge. For patients, prominent factors included the misunderstanding of medical behavior and high expectations for prognosis (10).

This study not only identified the key risk factors for doctor-patient conflicts but also ranked their relative importance. The findings clearly indicate that “soft” communication and psychological factors (e.g., distrust and perceived neglect) are more critical predictors of conflict than “hard” clinical factors (e.g., a history of physical or mental disorders). Consequently, for emergency care, targeted efforts should focus on improving communication, managing patient expectations, mitigating the perception of prolonged waiting, and training healthcare staff in effective emotional de-escalation techniques.

Establishing a prediction model for medical conflict in emergency departments is conducive to preventing medical conflict, improving the work environment of medical professionals, and safeguarding the legitimate rights and interests of both doctors and patients. This study included 630 patients in the emergency department. We constructed a prediction model by screening the risk factors of medical conflict through neural network analyses. The prediction model for medical conflict in emergency departments established in this study demonstrated good predictive performance in internal validation, and a good cross-validated predictive accuracy in an independent holdout sample. Based on this model, emergency medical professionals can identify the risk factors and prevent conflict by accurately judging the patient’s condition classification, minimizing their waiting time, and giving appropriate rescue measures. In addition, they should pay attention to the emotional status of patients with a history of psychosomatic diseases, strengthen communication with patients and caregivers, and make efforts to meet patients’ reasonable needs as much as possible and promptly. They can also provide a detailed explanation of the treatment methods, care measures, and medical process to patients and caregivers and appropriately limit the number of caregivers, thereby reducing the incidence of medical conflict.

## 5 Limitations

Based on the characteristics of medical conflict in emergency departments, this study developed a prediction model for medical conflict in emergency departments, thereby filling the existing research gap. However, it is not without limitations. This study is a single-center study, and the research sample has certain limitations. We will make the exploration of prospective, multicenter studies a key focus of our subsequent research. We call for future studies to adopt more standardized methodologies to facilitate cross-national and cross-cultural comparisons.

## 6 Conclusion

Leveraging neural network algorithms, this study developed a highly efficient predictive model for assessing the risk of doctor-patient conflict in emergency settings. The model demonstrated exceptional predictive accuracy (AUC = 0.966), successfully pinpointing “patient distrust” and “prolonged waiting time” as the two most significant risk factors. These findings suggest that hospital administration should prioritize enhancing doctor-patient communication and streamlining emergency workflows. Furthermore, the model can be utilized to provide early warnings for high-risk situations, which can be applied to medical conflict early warning in emergency situations. The model has excellent predictive value. It can help medical professionals in emergency departments identify risk factors in a timely manner, guide appropriate clinical interventions, and reduce the incidence of medical conflicts in emergency departments. It has significant clinical value and practical applications.

## Data Availability

The original contributions presented in the study are included in the article/Supplementary material, further inquiries can be directed to the corresponding author/s.
