# Supplementary material for: A risk prediction model for medical conflict in emergency departments
Source: Front Public Health. 2026 Jan 29;14:1734894. doi: 10.3389/fpubh.2026.1734894 (PMC12894383; doi:10.3389/fpubh.2026.1734894)
Supplement: Supplementary file 1 [file Supplementary_file_1.docx]

Electronic supplementary files (ESF)

ESF, Table 1. Neural networks parameter estimates


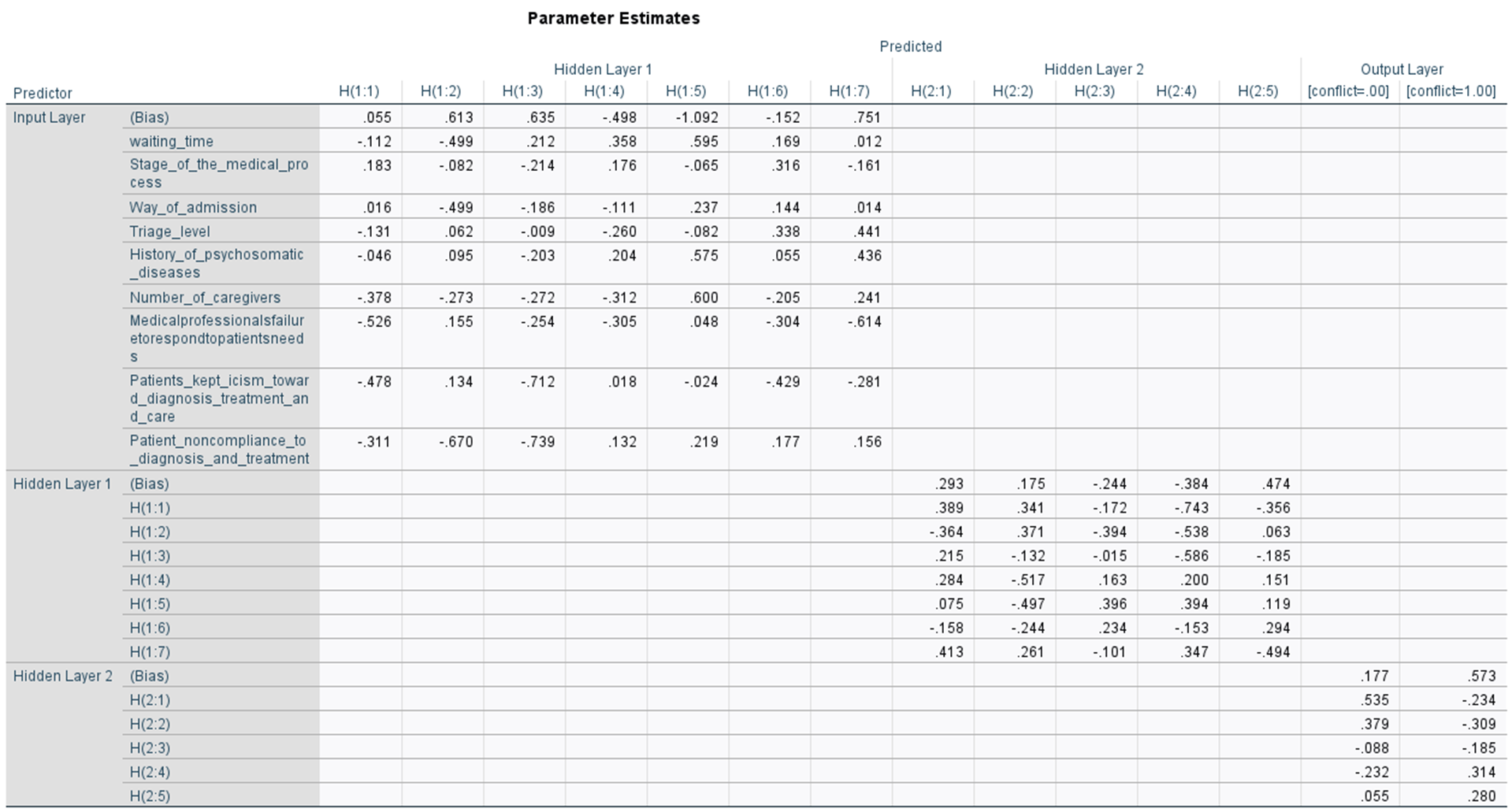


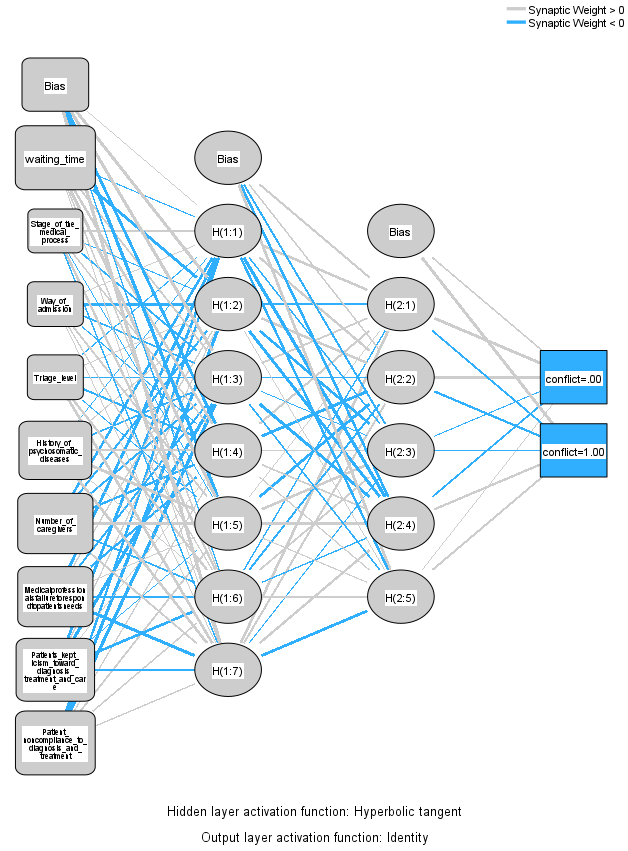


ESF, Figure 1. Neural networks diagram

ESF, Table 2. Neural network model summary


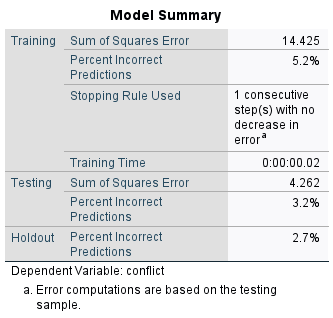


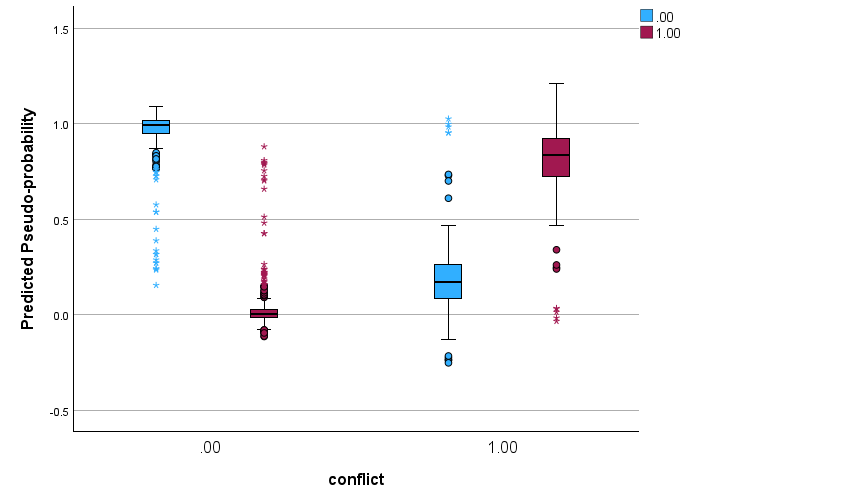


ESF, Figure 2. Neural networks predicted pseudo-probability versus observed probability.
